# Yellow Mustard Protein a Immunoreactivity Reduction Through Seed Germination, Lactic Acid Fermentation, and Cooking

**DOI:** 10.3390/foods13213498

**Published:** 2024-10-31

**Authors:** Simran Kaur Jawanda, Hosahalli S. Ramaswamy

**Affiliations:** Department of Food Science, McGill University, Macdonald Campus, 21111 Lakeshore Road, Ste Anne de Bellevue, QC H9X 3V9, Canada; simran.jawanda@mail.mcgill

**Keywords:** mustard, proteins, germination, fermentation, immunoreactivity

## Abstract

Food allergens are becoming increasingly threatening and are disrupting the health and social structure of a significantly large population worldwide. Proteins from mustard are among the well-recognized food allergens which affect many sensitive individuals. Many processing methods are continually being explored to reduce allergen immunoreactivity and for developing hypoallergenic foods. Cooking, germination, and fermentation have been evaluated to attenuate the immunoreactivity of food allergens. The objective of this study is to evaluate the effect of seed germination, lactic acid fermentation, and/or cooking on yellow mustard seed protein immunoreactivity (IR) (protein A) using ELISA techniques. Samples from five-day germination at 35–40 °C and three-day fermentation between 25 °C and 35 °C were evaluated. The germination and fermentation processes yielded varying reductions in the IRs of mustard proteins, with a combined yield of about 90% reduction. When complemented with further stovetop cooking, protein IR reduction was extended up to 98%, while cooking alone resulted only in about a 70% reduction. FTIR results confirmed that changes in mustard protein conformation maybe due to the unfolding and/or denaturation of mustard proteins. These processing methods are beneficial as they not only help reduce the native mustard protein IR, but also increased inherent antioxidant activities in germinated and fermented mustard seeds.

## 1. Introduction

Mustard is used in the preparation of various food products such as processed meats, salad dressings, seasoning blends, sauces, condiments, and pickled products in order to enhance its flavor and nutritional values [1]. Mustard seeds are the main ingredient of North American-style mustards. Mustard seeds are also major ingredients in Chinese and European-style mustards [2]. Mustard is recently added to the list of priority allergens in the European Union, as well as in Canada [3] (World Health Organization, 2010). Mustard allergy cases are reported to cause allergic symptoms and reactions such as oral allergy syndrome, immediate skin response, and more severe reactions such as anaphylactic shock in hyper-sensitive persons [4]. There is no successful treatment for allergy remediation, and avoiding products containing mustard is the only real solution to avoid allergic reactions in sensitive individuals. Known yellow mustard allergens associated with allergic reactions are Sin a 1, Sin a 2, Sin a 3, and Sin a 4. Out of these, Sin a 1 and Sin a 2 are major allergens characterized as seed storage proteins belonging to the 2S albumin and 11S globulin family, respectively [5,6], whereas Sin a 3 and Sin a 4 are a non-specific lipid transfer protein (nsLTP) and a profilin, respectively [7].

Food processing results in matrix interaction, structural changes, and/or solubility changes, and may have effect on the allergenicity of the final product [8]. Processing can affect allergic proteins and result in a decrease, increase, or no change in the immunoglobulin E (IgE) binding capacity of the allergen [9]. Food processing methods, including thermal and non-thermal treatments and type of treatment, may differ in their effects on epitopes. Thermal processing may be achieved using dry heat (for, e.g., roasting, frying, infrared heating) or using wet heat (for, e.g., autoclaving, boiling blanching, pressure cooking, ohmic heating, MW/RF heating, extrusion). Non-thermal processing includes high-pressure processing, grinding, milling, and dehulling and dehusking, to name a few [10]. Processing may result in alteration that permits the unmasking or masking of allergenic epitopes which may reduce, enhance, or have no effect on the allergenicity of the offending food. Some treatments like biological, biochemical, or chemical applications may influence the degree of immunoreactivity (IR). These methods include treating with additives, brining, curing, enzymatic treatment, fermentation, germination, pickling, and salting [11]. However, there has been no effective method to eliminate the allergenic capacity of mustard. In a recent study [12], the reduction in allergen immunoreactivity of mustard proteins was discussed through cooking and intense thermal processing treatments, indicating that, while conventional cooking resulted in only about a 60–80% reduction in the allergen IR, enhanced thermal processing (more severe than conventional commercial sterilization approaches) could result in 3–4 logarithmic reductions in the IRs of mustard allergens. Developing new minimal processing approaches to modify allergen content will have a significant additional advantage.

Recently, several studies have demonstrated that, during germination, the seed storage proteins, including allergenic proteins, are broken down into peptides or amino acids by catalytic enzymes that are responsible for providing the nitrogen required for seedling growth [13,14]. Depending on the specificity of the enzymes and epitopes’ susceptibility to the active enzymes, germination may result in the elimination of certain epitopes in seed storage proteins during germination period [15]. There are reported findings on rice and soybeans that show significant degradations in storage protein and reductions in immunoreactivity after short-term germination [12]. Traditionally, legume sprouts are very popular in eastern countries and are becoming increasingly acceptable to western countries’ consumers. There are a number of studies demonstrating that sprouts are novel functional foods and can act as dietary source of phenolic substances [16]. With short-term germination, the antioxidant polyphenols increase in legume seeds [17]. Recently, Assou et al. [18] demonstrated that the mustard seed germination process could be used to reduce allergens through genetic studies.

Furthermore, scientific information on the effects of fermentation on food allergens is very limited. Fermentation is considered to be a method to decrease immunoreactivity, as demonstrated by studies conducted on fermenting soybean, skim milk, and whey proteins [19]. Lactic acid fermentation can decrease the immunoreactivity of soya, and it has the potential to develop nutritious hypoallergenic soya products [20,21]. Combined strains of *Lactobacillus helveticus* and *Streptococcus thermophilus* were the most effective in reducing the antigenicity of whey proteins [22]. However, fermentation is a natural process which not only results in lowering immunoreactivity, but also improves the antioxidant and total phenol content of food products [23]. Verhoeckx et al. [9] indicated that different food processing techniques could be used to reduce allergen concerns. The application of one single processing technique may not show a significant reduction in the immunoreactivity of allergic foods. A combination between various processing techniques can provide a new strategy for decreasing immunoreactivity [24]. Research studies focusing on allergy reduction in mustard proteins using nonthermal methods are very scarce or are in their very early stages.

An enzyme-linked immunosorbent assay (ELISA) was used for allergen detection and quantification, as it is a very sensitive, simple, rapid, and accurate method [25]. A sandwich ELISA was employed for the identification and quantification of allergic proteins [26]. Additionally, changes in protein structure were studied using Fourier-transform infrared spectroscopy (FTIR). The Amide I region of the infrared spectrum was evaluated for protein secondary structure changes and was used to understand deviations in secondary structure such as conformational changes, that is, the unfolding and/or folding of protein and formation of aggregates [27]. Presently, there is very limited work published on demonstrating the effect of food processing technologies on yellow mustard proteins. Published information on qualitative changes is abundant on the denaturation and alteration of functional properties, but studies on quantifying the allergens in yellow mustard are almost nonexistent. Moreover, the relationship between the IRs and structural properties of mustard proteins has been not explored in detail. Fourier-transform infrared spectroscopy (FTIR) is a useful technique to provide information on the effects of germination and fermentation on the secondary structure of mustard proteins which can be related to the IR changes in mustard proteins.

Therefore, the aim of this study was to first track the reduction in immunoreactivity (IR) of mustard proteins through the non-thermal processing approach of seed germination and lactic acid fermentation of yellow mustard seeds. The second objective was to use FTIR to understand the conformational changes induced with the biological methods (germination and fermentation) and their correlation with the observed IR values, and finally to investigate the effect of these processing treatments on the antioxidant capacities of yellow mustard seeds.

## 2. Materials and Methods

### 2.1. Mustard Samples

Yellow mustard seeds (*Sinapis alba*) were purchased from Food to Live Company (Brooklyn, NY, USA).

### 2.2. Seed Germination

The experimental samples were prepared according to the method adapted from [28]. In total, 25 g of yellow mustard seeds were soaked in 50 mL of water for 24 h at 25 °C. Then, the seeds were weighed, tossed, and spread on plastic trays. Mustard seeds on each tray were incubated within the germinator (Kikiheim automatic bean sprouts machine, 25.5 cm × 34 cm, and tray aperture—1 mm with 2-layer germination tray) in the dark at a temperature in the range from 35 °C to 40 °C, controlled using the germinator’s operational settings, and 90% relative humidity for 0 to 5 day(s) of germination. All of the samples were ground and freeze-dried at −50 °C in a freeze-drier (Labconco Corporation, Kansas City, MO, USA) for further analysis.

### 2.3. Lactic Acid Fermentation

Preparation of the experimental sample was carried out with the solid-state fermentation of mustard seeds [21]. Raw mustard seeds were suspended in sterile distilled water (1:1, *w*/*v*) and kosher salt was added at a ratio of 1:25 (*w*/*w*). Furthermore, a mixed culture of active LAB strains, i.e., L. *plantarum*, Ln. *mesenteroides*, and Pc. *acidilactici* (2.4 × 10^8^ CFU/g), starter culture (Starter kit from Cutting Edge Cultures LLC, Wakefield, RI, USA), was inoculated at 0.1% (*w*/*w*) in raw seeds. This kind of fermentation is used to prepare European-style mustards and traditional fermented food in Taiwan [3,29]. The fermenting samples were incubated at 25 °C and 35 °C with a relative humidity of 90%, and were fermented from 1 to 3 day(s). All of the samples were then ground in a pestle mortar and freeze-dried at −50 °C in a freeze-drier (Labconco corporation, Kansas City, MO, USA) for further analysis.

### 2.4. Sandwich ELISA

A sandwich ELISA kit (3M Mustard Protein ELISA Kit, Maplewood, MN, USA) was used for the detection and quantitative analysis of mustard protein A groups. The employed sandwich ELISA used two matching antibody pairs (detection and capture antibodies). This ELISA kit contained a microtiter plate coated with an anti-mustard antibody and reagents including mustard protein standard concentrate, mustard horseradish peroxidase (HRP) conjugate, an extraction buffer, diluent solution, wash solution, chromogenic substrate solution, and stop solution. Mustard proteins present in the test samples were extracted with the extraction buffer, and they reacted with the anti-mustard antibody that is adsorbed onto the surface of polystyrene microtiter wells. The removal of unbounded fractions was carried out by washing them with a wash solution and anti-mustard antibodies conjugated with horseradish peroxidase (HRP) prepared with diluent solution. These enzymes, labeled as antibodies, form complexes with previously bound mustard proteins. This was followed by a second washing step, and then the enzyme bound to immunosorbent was detected by adding a chromogenic substrate, 3,3′,5,5′-tetramethylbenzidine (TMB), and then a stop solution was added to stop the reactions. Color was developed with the enzymatic reaction, and it varied directly with the concentration of absorbed mustard protein in the sample tested. This procedure has been detailed in [1]. Then, the final absorbance of mustard protein in each test sample was recorded at 450 nm as a measure of the concentration of mustard protein in the test sample. The concentration of mustard protein was calculated according to the standard curve obtained by spiking different concentrations of supplied standards in the kit in the same manner and reading the absorbance.

### 2.5. Fourier Transform Infrared Spectroscopy (FTIR)

Fourier-transform infrared spectroscopy (FTIR) was used to study the changes in secondary structure present in mustard protein. The freeze-dried sample (0.1 g) was transferred to the diamond crystal at room temperature. The data were analyzed using the Windows-based OMNIC software (Version 9, thermo Nicolet Co., Ltd., Madison, WI, USA) using a Nicolet™ iS50 FTIR Spectrometer with an ATR accessory (Nicolet Co., Ltd., Madison, WI, USA) equipped with a computer. A total of 128 scans of FTIR spectra at a resolution of 4 cm^−1^ were recorded and averaged in the mid-infrared region (4000–500 cm^−1^). To avoid the influence of air, a background spectrum without the sample was also collected before each determination. The spectra were deconvoluted and were used for calculating the percentage of the secondary structure in the Amide I region of mustard protein with a bandwidth of 31 cm^−1^ and an enhancement factor of 2.4 [30].

### 2.6. Conventional Cooking of Treated Samples

In order to compare them conventional cooking, selected germinated and fermented samples were freeze-dried, and the freeze-dried flours were used to prepare a 5% slurry by mixing selected freeze-dried samples with double-distilled water. These samples were also cooked in boiling water at 100 °C for 30 and 60 min to evaluate the effect of cooking under atmospheric processing conditions. The slurry sample was filtered through Whatman #4 filter paper, and the clear extract was analyzed. As mentioned earlier, previous studies [12] indicated that both conventional cooking and intense thermal processing significantly enhance IR reduction in mustards. Cooking itself accounted for about a 60–90% reduction in mustard IR. Hence, this was used for comparison in this study, both on its own merit as well as an additional treatment following sonication/fermentation.

### 2.7. Phenolic Compounds and Antioxidant Activity

#### 2.7.1. Extraction of Phenolic Compounds

The approach to extract phenolic compounds was adapted from the method outlined by Marathe et al. [31]. In total, 0.1 g of raw and freeze-dried germinated and/or fermented samples were dissolved in 10 mL of 100% methanol (Millipore Sigma Canada Ltd., Oakville, ON, Canada). Then, these samples were placed in a shaking water bath (Julabo USA, Inc., 884 Marcon Boulevard, Allentown, PA, USA) at 100 rpm and were incubated for 2.5 h at room temperature (28.2 °C). Afterwards, the mixture was centrifuged at 3000× *g* for 15 min. The supernatant was filtered and used for the antioxidant scavenging activity and total phenolic content assays.

#### 2.7.2. DPPH Antioxidant Scavenging Activity

The free-radical scavenging activity of all of the samples was determined using the DPPH (2,2-diphenyl-1-picrylhydrazyl) method described by Peters et al. [32]. A fresh solution of DPPH (0.1 mM) in methanol was first prepared. In total, 0.2 mL of methanolic extract of each sample was added to 3 mL of DPPH methanol solution. The sample solutions were vortexed and left to incubate at room temperature for 30 min in the dark. Thereafter, the absorbance value was measured at 517 nm by using a UV/VIS spectrophotometer (VWR, Model V-3100PC, West Chester, PA, USA). The control for this assay was prepared by adding 0.2 mL methanol to 3 mL of DPPH. The assay was performed in triplicate. The percentage scavenging activity was calculated using the following equation:

$$\%\;\mathrm{Scavenging}\;\mathrm{activity}=\frac{Abs\left(Control\right)-Abs\left(Sample\right)}{Abs\left(Control\right)}\times 100$$

where Abs (Control) is the absorbance of DPPH solution in methanol, and Abs (Sample) is the absorbance of DPPH solution mixed with the sample.

#### 2.7.3. Total Phenol Content

The determination of total phenol content was based on the Folin–Ciocalteu method [33]. Initially, 0.5 mL of methanol was added to 0.5 mL of methanolic sample extract. Subsequently, 5 mL of Folin–Ciocalteu reagent (diluted 10 times with double distilled water) and 5 mL of aqueous sodium carbonate solution (7.5%, *w*/*v*) was added to the reaction mixture. Afterwards, the sample solutions were vortexed and incubated for 20 min at room temperature. The absorbance value was measured at 760 nm against a blank (prepared in the same way, but replacing the sample with methanol). The standard curve range was 50–350 μg/mL gallic acid (R^2^ = 0.9988). The results were expressed as mg of gallic acid equivalent (GAE)/g dry weight (DW).

### 2.8. Statistical Analysis

The data were analyzed using one-way analysis of variance (ANOVA) followed by post hoc using Tukey’s test (*p* < 0.05) using SPSS 27.0 analytical software (SPSS Inc., Chicago, IL, USA). All treatments and experimental studies were conducted in triplicate.

## 3. Results

### 3.1. Germination Studies

#### 3.1.1. Mustard Protein Immunoreactivity (IR) Reduction

The immunoreactivity of mustard proteins were quantified by forming a standard curve by spiking mustard protein standard provided in the sandwich ELISA kit. Dilutions of samples were carried out to bring the concentration within the range established by the nonlinear standard curve, fitting a second-order polynomial (quadratic model). Allergen concentration was calculated from a standard curve in ppb (ng/L) and was multiplied with the dilutions factors [34]. This assay was performed to assess the effect of seed germination on IgE-binding capacity.

**Figure 1:**
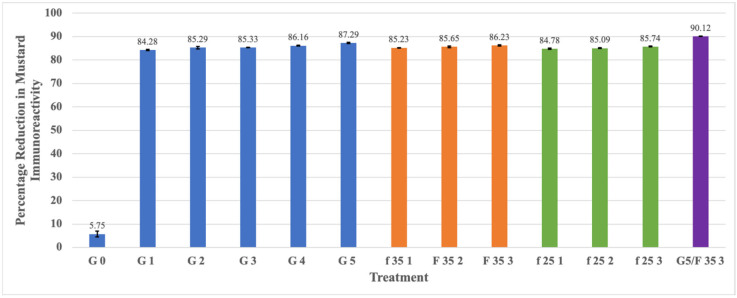
Percentage reduction in mustard protein immunoreactivity of germinated and fermented mustard seeds. The notations are detailed in Table 1 (G0–G5 for germination and F35,25 1–3 for fermentation); Different colors represent different groupings.

The results presented in Table 1 show that the IgE-binding capacity of mustard seeds continued to decline with the progress in germination. The IgE-binding capability started to decline rapidly after the first 24 h of soaking/germination. The mustard protein immunoreactivity decreased rapidly after 24 h germination, resulting in a decrease in an IgE reactivity-based native protein from 232 × 10^3^ to 38.7 × 10^3^ ppm (84% reduction), with only a gradual further reduction in the next five days of germination to 31.3 × 10^3^ ppm (87% reduction). This represented an overall 0.9 cumulative log reduction in protein immunoreactivity. The progressive percentage reduction in the mustard protein immunoreactivity of mustard seeds during the five-day germination process is shown in Figure 1. Therefore, the five-day germination of yellow mustard seeds was deemed to have a positive effect on IgE-binding capacity of mustard proteins. In a previous study on the effects of germination on the IgE-binding capability of peanuts, it was observed that IgE binding began to decrease after soaking and exhibited a downward trend during the first four days of germination, which was significant as compared to raw or soaked peanut samples [35]. Similar results on soybean germination were reported 72 h after seed germination in another study [14]. Since the IgE based immunoassay is specific to native mustard proteins and its reduction is, therefore indicative of some structural modifications/transformation of native proteins or their partial hydrolysis due to the enzymatic activity during the germination process. During the germination, proteases are activated, the internal metabolism becomes accelerated, and proteins are hydrolyzed.

#### 3.1.2. Fourier-Transform Infrared Spectroscopy of Thermally Processed Samples

**Figure 2:**
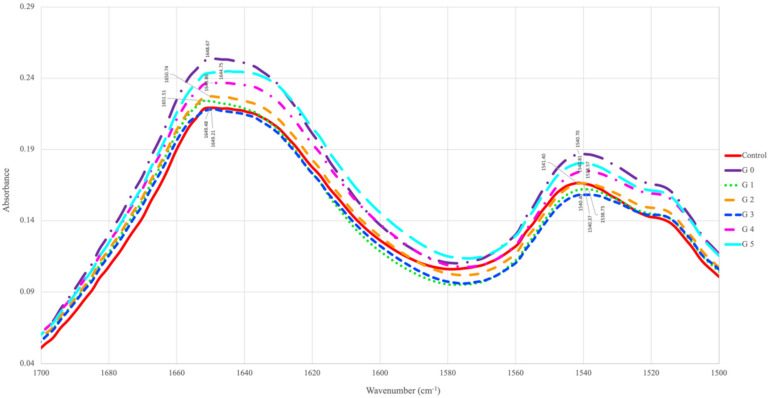
FTIR—spectra of Amide I and II components of raw and germinated mustard seeds. The notations are detailed in Table 1 (G0–G5 for germination).

Fourier-transform infrared spectrometry (FTIR) analysis is certainly one of the most important analytical techniques for studying conformational changes in the Amide I and Amide II regions of processed samples. The Amide I and Amide II regions are most important for interpreting the vibrational bands of protein backbones [36]. To study the protein secondary structure changes that are conformational changes (folding and/or unfolding of protein and formation of aggregates), Amide I region deviations in the infrared spectrum are used [27]. This region is associated with the C=O stretching of the peptide backbone and N-H bending vibrations [37]. Amide I comprises overlapping bands of a number of secondary structures that are *β*-sheets, *α*-helices, turns, and randomly coiled conformations [38]. Deconvolution of Amide I bands isolated each band and differentiated its frequency to determine the right secondary structure components and quantify them [39,40]. The Amide I region bands are assigned to β-sheets (1613–1637; 1682–1696), α-helices (1645–1662), turns (1662–1682, 1630), and unordered (1637–1645) [41]. The typical FTIR spectra of the Amide I and Amide II regions of raw and germinated mustard seeds are shown in Figure 2.

**Figure 3:**
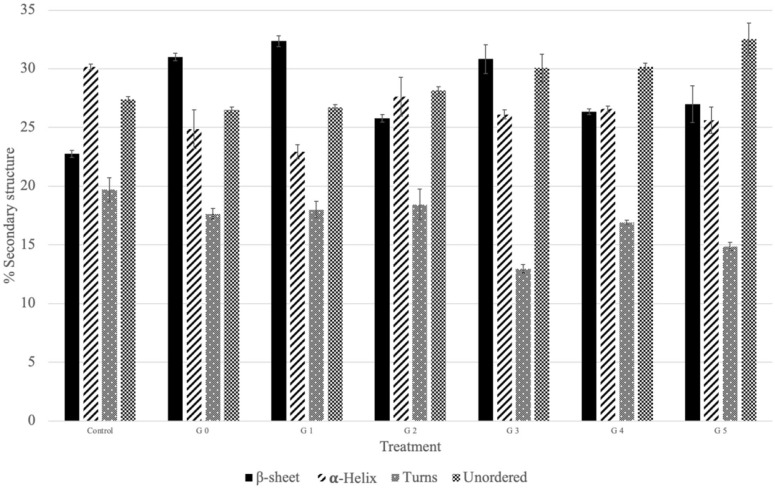
FTIR—secondary structure of raw and germinated mustard seeds. The notations are detailed in Table 1 (G0–G5 for germination).

The FTIR spectra band observed at 1700–1500 cm^−1^ for raw and germinated mustard seed samples corresponded to the Amide I and Amide II groups of proteins. The vibrations observed for mustard samples showed slight variation in wavenumber in germinated mustard seeds samples as compared to raw mustard samples. This could be due to the unfolding and/or denaturation of proteins and modifications in the protein chain during the germination process. Similar observations were also reported in [42], the wavenumber shifts were observed in the amide region for germinated chickpea flour when compared to native flour. These slight differences observed in the protein region (1700–1600 and 1545–1535 cm^−1^) corresponds to protein degradation during processing treatments [43]. The changes occurring in the secondary structures of mustard proteins when undergoing the germination process are shown in Figure 3. It was observed that the percentage of β-sheets and unordered structures increased with the five-day germination of raw mustard seeds, whereas reductions in α-helices and turns percentages were recorded. These findings suggest that the germination process is responsible for the secondary structure changes in the Amide I region. Similar results were observed in [44] in the case of germinating cereal grains. These results confirm that there exists some unfolding/denaturation or secondary structure changes due to the germinating process.

### 3.2. Fermentation Studies

#### 3.2.1. Mustard Protein Immunoreactivity Reduction

Table 1 also shows that mustard protein content in fermented mustard seeds decreased rapidly after first day of fermentation and declined slowly in next stages of fermentation both at 25 °C and 35 °C. It was observed that 3 days of mustard seed fermentation decreased the protein immunoreactivity by approximately more than three-fourths, as compared to that at 0 day. The concentration of mustard protein was lower when mustard seeds were fermented at 35 °C than when they were fermented at 25 °C. In the control samples with mustard protein concentration values of 246 × 10^3^ ppm, the value reduced to 33.9 × 10^3^ ppm when fermented at 35 °C for 3 days and 35.1 × 10^3^ ppm when fermented at 25 °C for 3 days, representing 0.86 and 0.85 log reductions, respectively. These results suggest that a significant reduction (*p* < 0.05) in immunoreactivity is possible due to fermentation and also demonstrate the efficacy of mixed culture to reduce mustard protein immunoreactivity by more than 84%, as demonstrated in Figure 1. According to [20], fermented soyabeans have significantly reduced immunoreactivity because of their induced fermentation. This study also suggests that L. plantarum culture exhibits a better potential for the development of reduced immunoreactive fermented soyabean as compared to mold strains. In another study, both natural and induced fermentation results showed a reduced IgE-binding capacity of nearly 89% in soybean meal [21]. Another previous study [45] also suggest LAB’s potential to reduce antigenic response in whey and skim milk. The additional synergic effects of immunoreaction reduction were demonstrated when a mixture of LAB and S. thermophilus strains were used. The combined strains’ effect on whey proteins during fermentation was also observed in [22], and also demonstrated that fermentation with lactic acid bacteria was an effective way to decrease the antigenicity of whey proteins. Again, as with the germination, the fermentation process also could result in the structural modifications/transformation of native proteins or partial proteolysis by fermenting enzymes.

#### 3.2.2. FTIR Results

**Figure 4:**
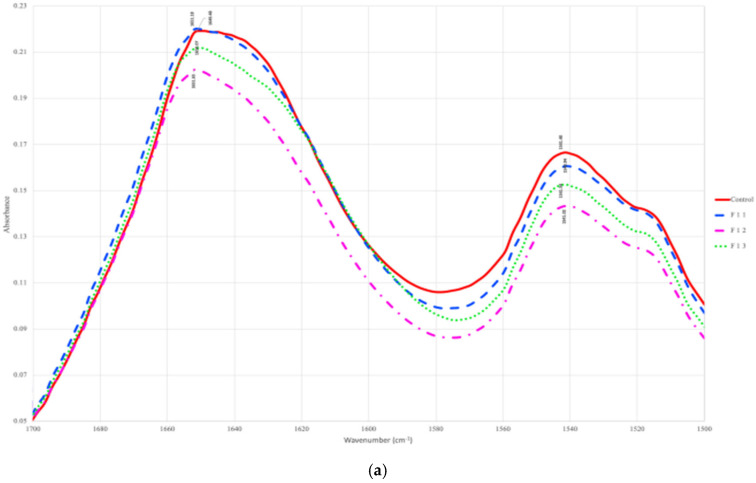

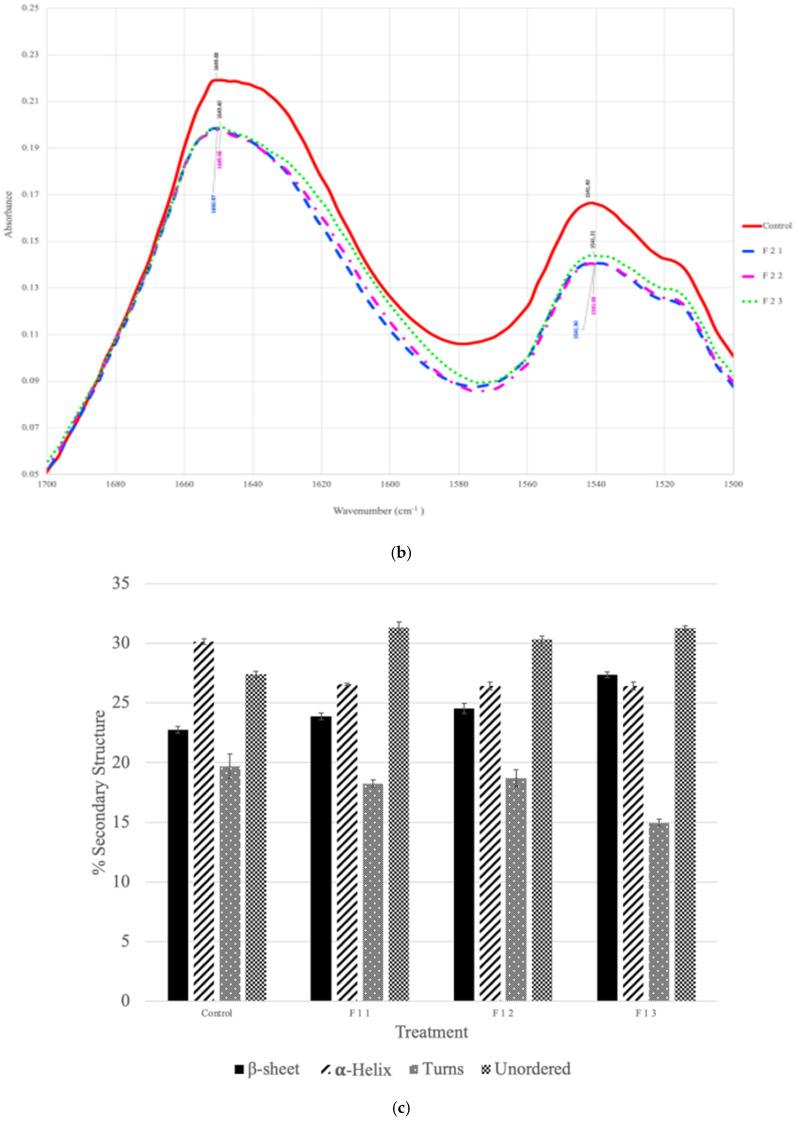

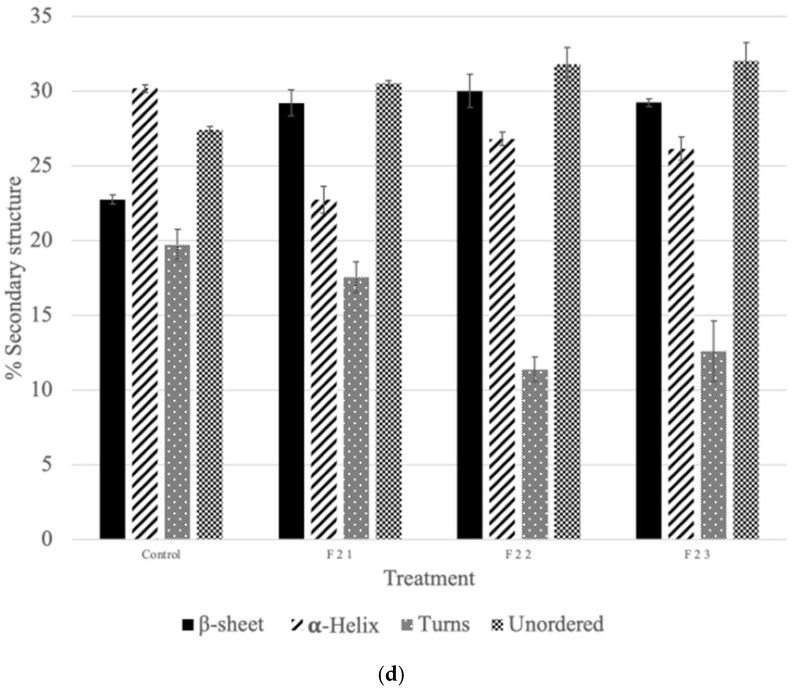
FTIR-spectra of Amide I and Amide II components of raw and fermented (35 °C) mustard seeds (**a**), raw and fermented (25 °C) mustard seeds (**b**), secondary structure in raw and fermented (35 °C) mustard seeds (**c**), raw and fermented (25 °C) mustard seeds (**d**). The notations are detailed in Table 1 (F35,25 1–3 for fermentation).

In the case of fermentation, minor shifts in wavenumbers (cm^−1^) were observed in both fermenting mustard seeds at 35 °C and 25 °C, as shown in Figure 4a and Figure 4b, respectively. Wavenumber variation in fermentation duration (day 1–3) was more prominent when fermented at 35 °C, whereas fermenting at 25 °C resulted in less-evident variations in wavenumbers from raw mustard samples. Therefore, fermenting mustard seeds at 35 °C using mixed-strain cultures was more beneficial than fermenting at 25 °C and resulted in more C=O stretching vibrations in the Amide I region. Similar changes in amide ide region were observed in [46], and the results also revealed a significant increase in protein content during fungal fermentation by A. oryzae. In a pervious study, it was observed that solid-state fermentation with A. niger resulted in increase in C=O stretching in amide ide groups in proteins of rapeseed meal [47]. In the case of fermented broccoli samples, the peaks between 1550 and 1640 cm^−1^ were associated with amide-stretching bands of proteins [48]. The fermentation process also resulted in similar secondary structure changes, as observed in the case of germinating mustard seeds and as demonstrated in Figure 4c,d. Three-day fermentation resulted in an increase in β-sheets and unordered structures and decreased percentages of α-helices and turns both in the cases of 25 °C and 35 °C. These results agree with a previous study [49] which showed the level of β-sheet structure increased in secondary structure of fermented horseradish sauce while α-helices percentage decreased when compared with unfermented samples. These results also confirm that there exists some unfolding/denaturation or secondary structure modifications due to the fermentation process.

### 3.3. Combination of Germination and Fermentation Treatments

#### 3.3.1. Mustard Protein Immunoreactivity Results

A remarkable reduction in detectable mustard protein was observed when the best combination of both processing techniques was combined, resulting in a final value of mustard protein concentration of 24.4 × 10^3^ ppm, decreased from 246 × 10^3^ ppm, which means one log reduction in protein immunoreactivity, as shown in Table 1. This means it resulted in a more than 90% reduction in immunoreactivity of mustard proteins, as is evident from Figure 1. The combined effect of both methods showed additional benefits in mustard protein immunoreactivity reduction than when applied separately. Therefore, the biological methods seem to be very effective and demonstrated the potential to explore such processing methods to reduce allergen immunoreactivity. The synergistic effect of the combination obviously results from the continuation of protein hydrolysis as a result of the two processes.

#### 3.3.2. FTIR Results

Figure 4a represents the FTIR spectra for the ecombined treatment of 5-day germination followed by fermentation at 35 °C for 3 days and raw mustard seed samples. Major variations in the Amide I and Amide II regions were observed when both germination and fermentation methods were applied to yellow mustard seeds. The secondary structure changes obtained from the FTIR results for mustard seeds samples treated with both processes, germination and fermentation, are shown in Figure 4b. Comparing the combined treatment samples that germination for 5 days followed by fermentation (35 °C) for 3 days resulted in the largest increase in percentages of α-helices and turns, and decreases in β-sheet and unordered structures. Therefore, the FTIR results show that the secondary structure of mustard proteins are sensitive to biological processing methods. These treatments are promising methods for bringing changes in the secondary structure of mustard proteins. Hence, the combination treatment is more beneficial than the individual one.

### 3.4. Conventional Cooking

The conventional cooking of the prepared 5% extract slurry of selected freeze-dried mustard samples showed reductions up to 0.48 ppm as compared to the control sample’s value of 26.6 ppm, as described in Table 2. In the case of 5-day germinated mustard samples, protein immunoreactivity reduced from 26.6 ppm to ~1 ppm after cooking for 60 min. Samples prepared from mustard seeds fermented for 3 days at 35 °C also showed a final protein immunoreactivity of 1.12 ppm after 60 min cooking. The highest reduction was observed for cooked samples prepared from the best combination of both biological methods, with the end value of mustard allergen concentration being 0.48 ppm after cooking for 60 min.

**Figure 5:**
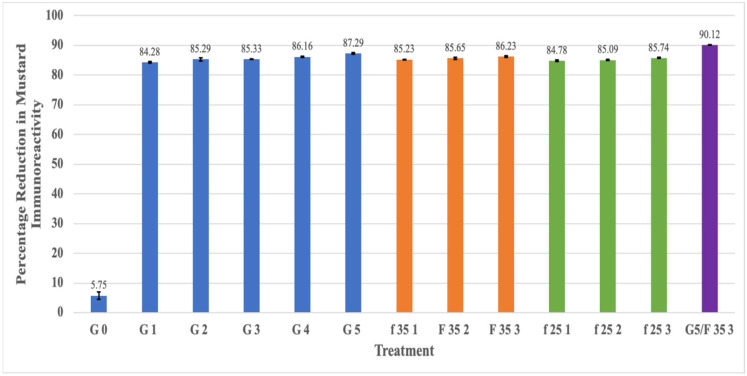
Percentage reduction in mustard protein immunoreactivity of cooked 5% extract slurry of selected germinated and fermented mustard seeds. The notations are detailed in Table 1 (G0–G5 for germination and F35,25 1–3 for fermentation); Different colors represent different grouping.

Considering the percentage reductions shown in Figure 5, the control samples treated for 30 min and 60 min resulted in approximately 41% and 68% reductions in protein immunoreactivity, respectively. This percentage reduction corresponds to a 0.23 and 0.50 log reduction in mustard protein immunoreactivity when cooked for 30 min and 60 min, respectively. For germinated mustard samples, the percentage reduction in protein immunoreactivity of cooked slurry samples ranged between 91% and 96%, approximately. Additionally, in this case, the highest log reduction was observed for the cooked slurry extract prepared from a germinated day 5 sample that had a 1.40 log reduction in mustard protein immunoreactivity. On the other hand, fermentation showed similar reduction with 3-day fermentation at 35 °C, showing a final protein immunoreactivity reduction at 95.8%, which translates to a 1.37 log reduction. Also, samples prepared from the best combination of both biological methods applied to thermal processing showed the highest reductions in mustard protein immunoreactivity, resulting in a 96.7% reduction in 30 min heating and a 98.2% reduction when heated for 60 min, as well as reaching log reductions of 1.48 and 1.74 in mustard protein immunoreactivity, respectively.

Therefore, it is evident that there is progressive reduction in protein immunoreactivity with both germination and fermentation durations, as well as with cooking. While the reduction in immunoreactivity during germination and fermentation can be assumed to result from structural transformation or enzymatic hydrolysis process, the one from cooking obviously results from the protein denaturation. Overall, the combination of these techniques could result in an impressive 98% reduction in immunoreactivity of mustard proteins, after combining it with a dilution factor based on percentage inclusion of the mustard ingredient in sample preparations. Prepared mustard sauces and such ingredients are added directly in small quantities to sandwiches. The use of germinated, fermented, cooked, and freeze-dried preparation methods in such processes would present much lower levels of allergy to active mustard proteins. Mustard seeds are added in spoon-sized quantities directly in many food preparations before cooking, baking, roasting, etc. Again, using germination–fermentation–cooked–freeze-dried preparation in such processes would have further varying effects on reducing mustard protein immunoreactivity.

Thermal treatment can cause modifications in protein structure due to denaturation, peptide bond hydrolysis, and aggregation by covalent and disulfide bonds, and the extent of the effect depends upon the duration and severity of processing [50]. Since these conventional cooking treatments can induce further changes in the denaturation of mustard proteins, they could lead to a lowering in general immunoreactivity intensities in treated samples.

### 3.5. Antioxidant and Total Phenolic Content

Considering the antioxidant activity, significant differences were observed in the percentage radical scavenging activity depending on the processing method applied to the yellow mustard seeds. It was observed that the sprouting resulted in an improved percentage radical scavenging activity, increasing from 24.2% to 32.1% after 5 days of germination. This implies that the ability of sprouted mustard seeds to scavenge free radicals increases after the germination process. The antioxidant properties of germinated black (*Brassica nigra*) and brown (*Brassica juncea*) mustard seeds were improved in a study [51]. In cases of fermenting mustard seeds, a higher DPPH antioxidant scavenging activity was observed as compared to their seed counterparts, as shown in Table 3. These are additional advantages that can be realized due to the germination and fermentation processes.

These processes, therefore, are promising with respect to reducing the immunoreactivity of mustard proteins, with the added benefit of enriching the antioxidant potential, thus providing a dual advantage. It is hoped that the observed trends in reduced IR that might eventually lead to possible reductions in allergen proteins in the short run, and, in the long run, possible reductiona in allergenicity.

## 4. Conclusions

The major conclusion of this study is that biological methods such as germination and fermentation are effective methods to decrease the immunoreactivity of protein in mustards seeds. The sandwich ELISA demonstrated that five-day germination resulted in an 87% reduction in mustard protein A immunoreactivity, which means a 0.90 log reduction. Similarly, an 86% or 0.86 log reduction in protein immunoreactivity was observed for fermentation at 35 °C. The alterations in mustard protein structure, as demonstrated with the FTIR results, may have led to unmasking, masking, or epitope destruction resulting in changes in immunogenic response, as observed in the ELISA protein immunoassay. The conventional cooking of 5% slurry extract of these treated samples at 100 °C demonstrated further reductions in protein immunoreactivity from 26.6 ppm to 0.480 ppm, which is an approximately 1.68 log reduction, and the percentage reduction in immunoreactivity was beyond 98%.

These processing methods are beneficial, as they also resulted in increased antioxidant activities in germinated and fermented mustard seeds. The use of such bio-processing methods should provide good incentives as non-thermal alternatives for food allergen reducing and antioxidant enhancing concepts for mustard processing, which, when further combined with other procedures such as cooking, result in further reductions in immunoreactivity in mustard allergens.

It should be noted that this study, and many other similar laboratory studies on the quantification of proteins, only provide some possible insights into the methods that can reduce allergen contents; however, they do not necessarily have any bearing on the allergenicity of the samples. Different people may have different levels of allergic reactions to different allergens. Some may be sensitive even at ppm or ppb levels, while others may remain unaffected even when their levels are large.

These studies provide guidelines as to which processing methods can potentially reduce the allergen contents in the samples and possibly might elicit reduced symptoms. Only animal or clinical trials can reveal the real influence of these allergens on individuals. It is recommended that future animal or human clinical studies take notice of these methods which can potentially reduce the levels immunoreactive proteins and use these products in their studies to test the efficacy of allergenicity reduction. It is hoped that the products suggested in this study, or the ones from the earlier intense thermal processing procedures [12], can help to reduce the incidence of mustard allergenicity in consumers.

Furthermore, many studies do use these types of ELISA techniques for quantification allergens and reductions in allergenicity. This is incorrect. We have used the term immunoreactivity of allergen proteins in some previous studies. But we should still be more specific as to what the ELISA kit refers to—in this case, murtad A proteins. Hence, we have stressed that this study refers to the immunoreactivity of mustard A proteins, but may have a relationship with mustard allergens as well. From a testing point of view, any residual unmodified protein from an ingredient is indicative of allergen risk from that ingredient and is hence notified.

## Figures and Tables

**Table 1:** Mustard allergen concentration of germinated and fermented mustard allergens.

Type of Treatment	Temperature (°C)	Time Day(s)	Mustard Protein Concentration (ppm)	Logarithmic Reduction in IR [log(10)]
Control	-	-	246 × 10^3^ ± 1.42 ^a^	0.00
Germination (G)	35–40	0	232 × 10^3^ ± 1.81 ^b^	0.03
		1	38.7 × 10^3^ ± 0.54 ^c^	0.80
		2	36.3 × 10^3^ ± 1.31 ^cde^	0.83
		3	36.1 × 10^3^ ± 0.25 ^cde^	0.83
		4	34.1 × 10^3^ ± 0.46 ^cef^	0.86
		5	31.3 × 10^3^ ± 0.33 ^ef^	0.90
Fermentation (F)	35	1	36.4 × 10^3^ ± 0.37 ^cde^	0.83
		2	35.4 × 10^3^ ± 0.55 ^de^	0.84
		3	33.9 × 10^3^ ± 0.32 ^ef^	0.86
	25	1	37.5 × 10^3^ ± 0.25 ^cd^	0.82
		2	36.7 × 10^3^ ± 0.27 ^cde^	0.83
		3	35.1 × 10^3^ ± 0.22 ^de^	0.85
G and F	G (35–40), F (35)	G-5, F-3	24.4 × 10^3^ ± 0.21 ^g^	1.01

Data are presented as mean ± SD of three independent observations. The mean difference is significant at the 0.05 level. Different superscript letters within a column indicate significant differences (*p* ˂ 0.05).

**Table 2:** Mustard protein concentration of conventionally cooked 5% slurry prepared from freeze-dried germinated and fermented mustard samples.

Type of Treatment	Temperature (°C)	Time (min)	Mustard Protein Concentration (ppm)	Reduction in IR [log(10)]
Control	-	-	26.6 ± 0.59 ^a^	0.00
Control	100	30	15.6 ± 0.2 ^b^	0.23
	100	60	8.5 ± 0.6 ^c^	0.50
Germination (day 4)	100	30	2.20 ± 0.0 ^d^	1.08
	100	60	1.93 ± 0.27 ^d^	1.14
Germination (day 5)	100	30	1.53 ± 0.18 ^ef^	1.24
	100	60	1.07 ± 0.11 ^fg^	1.40
Fermentation (35 °C) (day 2)	100	30	1.77 ± 0.06 ^de^	1.18
	100	60	1.23 ± 0.04 ^efg^	1.33
Fermentation (35 °C) (day 3)	100	30	1.57 ± 0.22 ^ef^	1.23
	100	60	1.12 ± 0.34 ^fg^	1.37
Germination (day 5) and Fermentation (35 °C) (day 3)	100	30	0.89 ± 0.05 ^gh^	1.48
	100	60	0.48 ± 0.12 ^h^	1.74

Data are presented as mean ± SD of three independent observations. The mean difference is significant at the 0.05 level. Different superscript letters within a column indicate significant differences (*p* ˂ 0.05).

**Table 3:** This table presents the percentage of radical scavenging activity and total phenolic content of methanolic extract of prepared mustard samples.

Type of Treatment	Temp (°C)	Time Day(s)	% Radical Scavenging Activity	Total Phenolic Content (mg GAE/g Sample)
Control	-	-	24.20 ± 0.24 ^a^	3.49 ± 0.01 ^a^
Germination (G)	35–40	0	28.59 ± 0.22 ^bc^	3.85 ± 0.02 ^b^
		1	29.16 ± 0.11 ^bcd^	5.85 ± 0.07 ^f^
		2	30.46 ± 0.08 ^def^	6.72 ± 0.05 ^g^
		3	31.48 ± 0.06 ^ef^	7.09 ± 0.02 ^h^
		4	31.89 ± 0.26 ^fg^	7.76 ± 0.03 ^i^
		5	32.05 ± 0.09 ^fg^	7.92 ± 0.02 ^i^
Fermentation (F)	35	1	28.62 ± 0.13 ^bc^	5.50 ± 0.01 ^e^
		2	29.89 ± 0.13 ^cde^	6.98 ± 0.04 ^h^
		3	30.69 ± 0.09 ^def^	7.04 ± 0.15 ^h^
	25	1	24.69 ± 0.03 ^a^	4.57 ± 0.10 ^c^
		2	25.68 ± 0.04 ^a^	5.08 ± 0.02 ^d^
		3	28.07 ± 1.96 ^b^	5.49 ± 0.06 ^e^
G and F	G (35–40), F (35)	G-5, F-3	33.15 ± 0.06 ^g^	8.16 ± 0.02 ^j^

Data are presented as mean ± SD of three independent observations. The mean difference is significant at the 0.05 level. Different superscript letters within a column indicate significant differences (*p* ˂ 0.05). - indicates control is not treated with germination or fermentation.

## Data Availability

The original contributions presented in the study are included in the article, further inquiries can be directed to the corresponding author.
